# Short-term outcomes of repair vs. replacement for rheumatic mitral valve disease

**DOI:** 10.3389/fcvm.2025.1635587

**Published:** 2025-08-15

**Authors:** Ming Hou, Wei Zhou, Ning Zhang

**Affiliations:** ^1^ Department of Cardiac Macrovascular Surgery, Affiliated Hospital of North Sichuan Medical College, Nanchong, Sichuan, China; ^2^ Department of Cardiothoracic Surgery, Dazhou Dachuan District People’s Hospital (Dazhou Third People’s Hospital), Dazhou, Sichuan, China

**Keywords:** rheumatic mitral valve disease, mitral valve repair, mitral valve replacement, hemodynamics, clinical efficacy

## Abstract

**Background:**

The main types of surgery for rheumatic mitral valve disease are traditional percutaneous mitral balloon valvuloplasty (PMBV), mitral valve replacement (MVR) with removal of the original valve, and valve repair with preservation of the original valve. Some studies have shown that mitral valve repair (MVr) has certain advantages compared with replacement.

**Methods:**

The clinical data of 166 patients with rheumatic mitral valve lesions admitted to the Department of Cardiac Macrovascular Surgery of the *Affiliated Hospital of North Sichuan Medical College* and the *Dazhou Third People's Hospital* were retrospectively analyzed to compare the hemodynamic changes after mitral valve repair and replacement.

**Results:**

Hemodynamic evaluation of MVr: (1) left ventricular end-diastolic diameters (LVEDD), left atrial end-systolic diameters (LAESD), mitral E-wave velocity, left ventricular ejection fraction (LVEF), mitral valve orifice area (MVOA), mitral pressure halving time (PHT), and mean pressure gradient (MPG) at each time point after MVr were improved compared with preoperative values (*P* < 0.05). (2) There was a significant improvement in the level of mitral regurgitation in MVr patients intraoperatively and at the time of discharge compared with preoperatively (*P* < 0.05). Hemodynamic evaluation of MVr and MVR: (1) Patients who underwent MVr had significantly lower LVEDD, LAESD, and mitral E-wave velocity than those of patients who underwent MVR at each postoperative time point (*P* < 0.05). (2) Patients who underwent MVr had lower left ventricular posterior wall thickness at end-diastole (LVPWd) than that of patients who underwent MVR at 3 and 6 months postoperatively (*P* < 0.05). (3) Patients who underwent MVr had lower LVEF than that of patients who underwent MVR at 6 months postoperatively (*P* < 0.05). (4) Patients who underwent MVr had lower left ventricular end-diastolic volume (LVEDV) than that of patients who underwent MVR at 3 months postoperatively (*P* < 0.05).

**Conclusions:**

Mitral valve repair and mitral valve replacement are effective in the treatment of patients with rheumatic mitral valve disease, with greater hemodynamic improvement after mitral valve repair than replacement and with greater short-term clinical efficacy than valve replacement.

## 1 Introduction

Rheumatic mitral valve disease is the most common form of valvular heart disease among cardiovascular diseases, including mitral stenosis (MS), mitral regurgitation (MR), and combined lesions, with high morbidity and mortality rates (1, 2). According to incomplete estimates, rheumatic mitral stenosis affects >40 million people worldwide and causes nearly 320,000 deaths annually, accounting for 1.6% of cardiovascular deaths, making it a major global health problem (3, 4). Currently, the main treatment modalities for rheumatic mitral valve disease are conventional percutaneous mitral balloon valvuloplasty (PMBV), mitral valve replacement (MVR) with removal of the original valve, and MVr with preservation of the original valve. Valve replacement is one of the conventional treatment modalities for rheumatic mitral valve disease, which mainly includes mechanical and bioprosthetic valves. Mechanical valves are associated with perivalvular leakage, infective endocarditis, and anticoagulation-related complications of prosthetic valves. Uchino et al. (5) followed up a 25-year study of patients with rheumatic heart disease (RHD) who underwent mechanical mitral valve replacement: the cumulative incidence of thromboembolism was 11%, and the incidence of intracranial hemorrhage, perivalvular leakage, and infective endocarditis increased over time. Bioprosthetic valves have the disadvantages of valve degradation and reoperation. Chen et al. (6) conducted a propensity score-matched study of 3,638 patients with rheumatic mitral valve disease. The results showed that patients who underwent bioprosthetic valve replacement had longer intensive care unit stays, longer hospitalizations, and higher 10-year reoperation rates. A study by Fu et al. (7) also found that in terms of postoperative all-cause mortality, the valve replacement group was three times as long as the molding group; in terms of postoperative valve-related complications, the valve replacement group was more than twice as long as the molding group, but in terms of postoperative reoperation rate, there was no significant difference between the two groups. MVr avoids the need for long-term anticoagulants, reduces anticoagulation-related complications, and also reduces the incidence of thromboembolic events, while restoring the normal anatomy and physiologic function of the mitral valve. In addition, MVr protects the autogenous valve and reduces the likelihood of perivalvular leakage and the incidence of valve-related adverse events. Dejsupa et al. (8) showed that patients who underwent MVr had lower mortality rates, higher 10-year survival rates, and lower rates of adverse events of valve-associated endocarditis. MVr is a relatively novel surgical concept and technique in recent years, both at home and abroad, and there have been few studies related to changes in hemodynamics after rheumatic mitral valve repair. Based on this, the present study was conducted to compare the hemodynamic indexes after MVr and MVR in patients with rheumatic mitral valvular lesions and to evaluate the short-term clinical efficacy of MVr, with a view to providing more choices for the clinical treatment of rheumatic mitral valve disease.

## 2 Materials and methods

This study is a retrospective study. From September 2020 to December 2023, approximately 212 patients with rheumatic mitral valve lesions underwent mitral valve replacement/repair at the Department of Cardiac Macrovascular Surgery of the *Affiliated Hospital of North Sichuan Medical College* and the *Dazhou Third People's Hospital*. According to the predefined inclusion/exclusion criteria, 166 patient medical records were ultimately enrolled in this study**.**

### 2.1 Research grouping

This study included a total of 166 patient medical records with rheumatic mitral valve disease, with 94 cases in the control group undergoing MVR and 72 cases in the observation group undergoing MVr.

### 2.2 Inclusion and exclusion criteria

#### 2.2.1 Inclusion criteria

Participants were included if they met all of the following: (1) met the diagnostic criteria (9) for rheumatic mitral valve disease in the “Chinese Expert Consensus on Standardized Echocardiographic Examination of Adult Heart Valve Disease”; (2) met the indications (10) for mitral valve surgery in the “Chinese Expert Consensus on Indications for the Surgical Treatment of rheumatic mitral valve disease”; 3. aged ≤60 years old; and (4) informed consent was obtained from both patients and their families prior to surgery.

#### 2.2.2 Exclusion criteria

Participants were excluded for any of the following reasons: (1) concurrent coronary artery bypass grafting, aortic valve replacement or repair, and tricuspid valve replacement or repair; (2) failure of mitral valve repair with intermediate conversion to MVR; (3) previous mitral valve surgery; (4) combined cardiac diseases such as cardiomyopathy and malignant arrhythmias; (5) comorbidities with other serious diseases such as coagulation dysfunction, severe hepatic or renal insufficiency; and (6) poor compliance, not regularly reviewed, or incomplete clinical data due to other reasons.

### 2.3 Surgical techniques

All surgical operations were completed by the same chief surgeon. Under general anesthesia, the patient was placed in a supine position, was disinfected, wore a towel, and routinely underwent transesophageal echocardiography (TEE). The median sternum was taken as the surgical incision, the sternum was split longitudinally, and heparinization was routinely performed at 3 mg/kg. The pericardium was cut in an inverted T-shape, pericardial adhesions were loosened, and the pericardium was suspended. Routine elevation of the aorta and superior vena cava catheterization was used to establish cardiopulmonary bypass. The aortic root was blocked, the myocardial protective fluid [domestic histidine–tryptophan–ketovalerate solution (HTK)] was perfused at the aortic root, ice chips were placed on the surface of the heart, and cardiac arrest was performed. Patients with combined atrial fibrillation underwent Cox-Maze IV surgery and left atrial appendage ligation according to the ablation pathway (11) in the “2023 KASNet Guidelines on Atrial Fibrillation Surgery.”

#### 2.3.1 Mitral valve replacement

The right atrium and atrial septum were incised, and the incision was extended toward the junction of the left atrium and the aortic root to form an “inverted T” or “L” joint incision (right atrium–atrial septum–left atrial roof joint approach). If a thrombus existed in the left atrium, it was removed first (patients with combined atrial fibrillation usually underwent Cox-Maze IV surgery and left atrial appendage ligation). The diseased mitral valve leaflets and tendon cords were excised, part of the posterior leaflets and tendon cords were preserved, and the cardiac chambers were flushed with physiological saline. The valve size was measured, followed by placement of interrupted mattress sutures around the mitral annulus. The mechanical heart valve (505DM, Medtronic, Inc.) was then implanted with all knots tied on the atrial side. After rewarming, the right atrium and atrial septal incision were closed, and the ascending aorta was opened. The patient was kept in a head-down position, a venting needle was left in the aortic root, and then the lungs were inflated to expel left heart gas. The heart resumed beating, and then a TEE was performed to assess prosthetic valve function. After stabilization of the circulation, mechanical ventilation was resumed, extracorporeal circulation was stopped, heparin was neutralized with ichthyoglobulin, and the arteriovenous cannula was removed. The atrial incision, aortic cannulation site, and sternotomy site were checked, and then a thorough hemostasis was achieved. The pericardium was sutured, a pericardial and mediastinal drainage tube was placed, the sternum was closed with interrupted stainless steel wires, and the muscle and subcutaneous tissues were sutured in layers.

#### 2.3.2 Mitral valve repair

**Figure 1:**
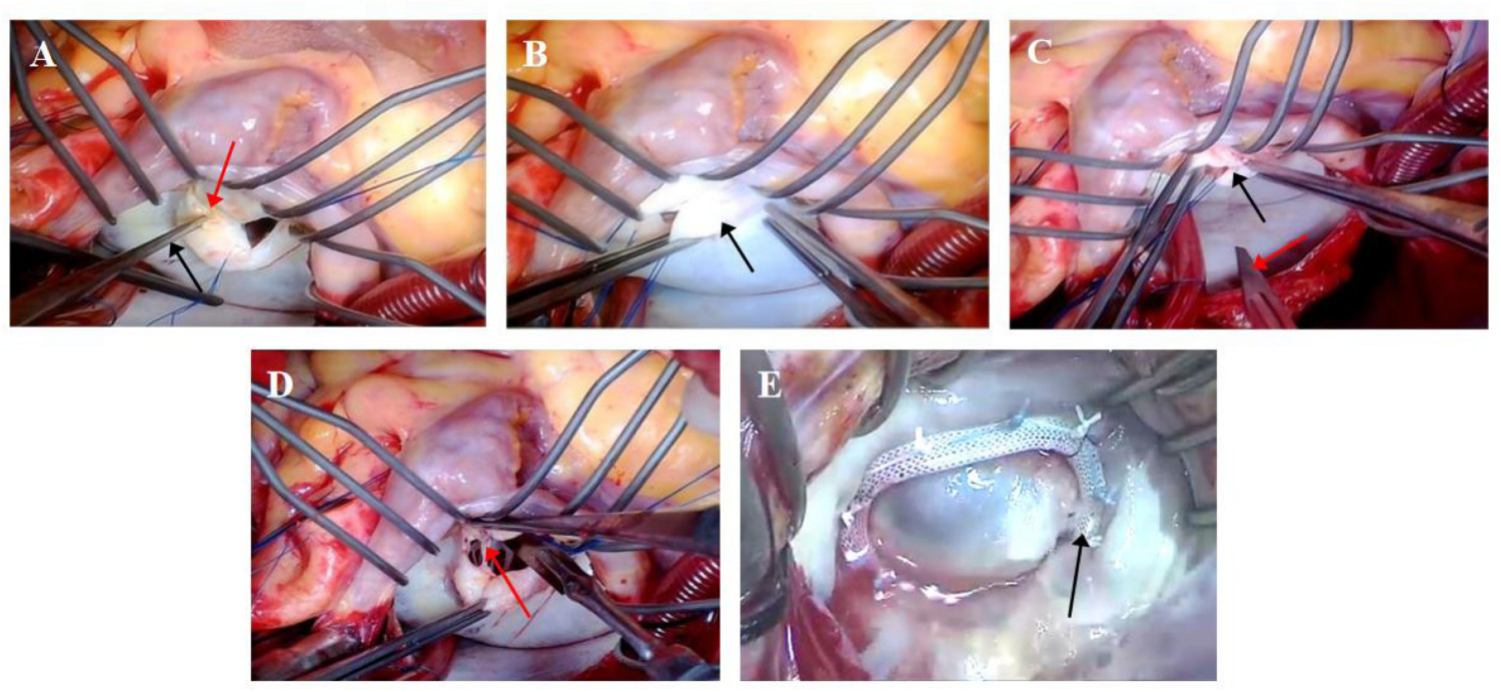
Surgical procedure for mitral valve repair [**(A)** debridement of calcified commissural plaques; **(B)** peeling of fibrotic leaflet plaques; **(C)** subcommissural incision and release; **(D)** lysis of subvalvular papillary muscle adhesions; **(E)** implantation of annuloplasty ring]. **(A)** Calcified plaques are indicated by red arrows, and the Penfield dissector is indicated by black arrows. **(B)** Fibrotic leaflet plaques are indicated by black arrows. **(C)** Valve commissures are indicated by black arrows, and the scalpel blade is indicated by a red arrow. **(D)** Chordae tendineae are indicated by a red arrow. **(E)** The annuloplasty ring is indicated by a black arrow.

The right atrium and atrial septum were incised, and the incision was extended toward the junction of the left atrium and the aortic root to form an “inverted T” or “L” joint incision (right atrium–atrial septum–left atrial roof joint approach). If a thrombus existed in the left atrium, it was removed first (patients with combined atrial fibrillation usually underwent Cox-Maze IV surgery and left atrial appendage ligation). A round knife and stripper (Freer elevator or Penfield dissector) were used to debride of calcified plaques at the mitral commissures (Figure 1A). The thickened fibrous tissue was stripped from the valve body (Figure 1B). Commissurotomy was performed along the mitral commissures, specifically opening the anterolateral and posteromedial commissures (Figure 1C). Micro-scissors or a round knife blade were utilized to lyse adhesions of the subvalvular papillary muscles (Figure 1D). The size of the mitral valve was measured, and a mitral valvuloplasty ring was implanted (Figure 1E). A physiological saline injection test was performed to evaluate mitral valve repair. After rewarming, the right atrium and atrial septal incision were closed, and the ascending aorta was opened. The patient was kept in a head-down position, a venting needle was left in the aortic root, and then the lungs were inflated to expel left heart gas. The heart resumed beating, and then a TEE was performed to assess valve function. After stabilization of the circulation, mechanical ventilation was resumed, extracorporeal circulation was stopped, heparin was neutralized with ichthyoglobulin, and the arteriovenous cannula was removed. The atrial incision, aortic cannulation site, and sternotomy site were checked, and then a thorough hemostasis was achieved. The pericardium was sutured, a pericardial and mediastinal drainage tube was placed, the sternum was closed with interrupted stainless steel wires, and the muscle and subcutaneous tissues were sutured in layers.

### 2.4 Postoperative management

1. For patients undergoing MVr, postoperative warfarin anticoagulation was maintained for 6 months. Patients receiving MVR were placed on lifelong warfarin therapy, with regular coagulation monitoring and dose adjustment based on international normalized ratio values.
2. All patients were scheduled for follow-up transthoracic echocardiography at 3 and 6 months postoperatively. These studies were performed and interpreted by the same certified echocardiography specialist to ensure consistency, with comprehensive recording of hemodynamic parameters.

### 2.5 Statistical analysis

All statistical analyses were performed with *SPSS* software (*version 26.0*; *SPSS Inc*., *Chicago, IL, USA*). Missing values were handled by the *K-nearest neighbor* algorithm. Categorical variables are expressed as frequencies and percentages. Continuous variables are expressed as the mean ± standard deviation. Median and interquartile range were used for non-normally distributed data. A two-tailed *P*-value of <0.05 was considered statistically significant.

1. A two-independent sample *t*-test was used for comparisons between groups for continuous variables, and the *Wilcoxon* test was used for non-normally distributed data.
2. Comparisons between groups for categorical variables were performed using the *χ*^2^ test (chi-square Pearson’s test for minimum expected counts *T* ≥ 5, chi-square continuity correction for 1 ≤ *T* < 5, and chi-square Fisher's exact method for *T* < 1).
3. Repeated-measures continuous variables were analyzed by repeated-measures *ANOVA*, and two-by-two comparisons at different time points were performed using the *LSD t*-test.

## 3 Results

### 3.1 Baseline characteristics

As demonstrated in Table 1, the two groups showed comparable baseline characteristics with no statistically significant differences in:

(1) Demographic parameters: age, height, weight, or sex distribution (all *P* > 0.05).
(2) Comorbidity profiles: prevalence of hypertension, diabetes mellitus, cerebral infarction, or coronary artery disease (all *P* > 0.05).
(3) Clinical status: New York Heart Association (NYHA) functional classification (*P* > 0.05).
(4) Additionally, the groups were well-matched in mitral valve pathology: distribution of mitral valve lesion types, severity of mitral stenosis, and degree of mitral regurgitation (all *P* > 0.05).
(5) Echocardiographic assessment revealed similar measurements for left ventricular end-diastolic diameters (LVEDD), LVEDD, left ventricular ejection fraction (LVEF), left ventricular end-diastolic volume (LVEDV), peak E-wave velocity, and left ventricular posterior wall thickness at end-diastole (LVPWd) (all *P* > 0.05).

**Table 1:** Comparison of baseline data between the two groups [$\bar{x}\pm s$, *n* (%), *M* (*P*_25_, *P*_75_)].

Variable	Control group (*n* = 94)	Observation group (*n* = 72)	*t*/*Z*/*χ*^2^ value	*P*-value
Age (years)	54 (49.25, 56.75)	51.94 ± 6.46	−0.906	>0.05^c^
Genders			0.381	>0.05^b^
Males	33 (35.10)	22 (30.60)		
Females	61 (64.90)	50 (69.40)		
Height (cm)	158.93 ± 7.88	159.17 ± 9.10	−0.183	>0.05^a^
Weight (kg)	58.83 ± 9.69	60.14 ± 8.36	−0.915	>0.05^a^
NYHA class			−1.917	>0.05^c^
I	0	0		
II	56 (59.58)	47 (65.30)		
III	37（39.36）	25 (34.70)		
IV	1 (1.06)	0		
Hypertension	5 (5.30)	6 (8.30)	0.211	>0.05^b^
Diabetes	5 (5.30)	6 (8.30)	0.211	>0.05^b^
Cerebral infarction	9 (9.60)	5 (6.90)	0.365	>0.05^b^
Coronary artery disease	1 (1.10)	1 (1.40)	-	>0.05^b^
Types of mitral valve lesions			−0.574	>0.05^b^
MS	49 (52.13)	39 (54.17)		
MS + MR	45 (47.87)	33 (45.83)		
MS severity			−0.443	>0.05^c^
Mild	10 (10.64)	10 (13.89)		
Moderate	33 (35.11)	28 (38.89)		
Severe	51 (54.25)	34 (47.22)		
MR severity			−1.17	>0.05^c^
None	42 (44.70)	34 (47.20)		
Mild	1 (1.10)	8 (11.10)		
Moderate	30 (31.90)	20 (27.80)		
Severe	21 (22.30)	10 (13.90)		
LVEDD (mm)	46.10 ± 5.17	45.81 ± 4.17	−1.926	>0.05^a^
LAESD (mm)	52.36 ± 6.25	49.42 ± 5.43	3.182	<0.05^a^
LVEF(%)	59.88 ± 7.20	57.89 ± 6.70	5.384	>0.05^a^
LVEDV (ml)	115.96 ± 31.01	107.87 ± 26.67	−1.803	>0.05^a^
E-wave velocity (m/s)	2.13 ± 0.49	2.03 ± 0.56	1.269	>0.05^a^
LVPWd (mm)	9 (8.10)	10 (9.10)	−1.03	>0.05^c^

^a^ *t-*test.

^b^ *χ*^2^ test

^c^ Wilcoxon test.

NYHA, New York Heart Association; MS, mitral stenosis; MR, mitral regurgitation; LVEDD, left ventricular end-diastolic diameter; LAESD, end-systolic diameter of left atrium; LVEF, left ventricular ejection fraction; LVEDV, left ventricular end-diastolic volume; LVPWd, left ventricular posterior wall thickness at end-diastole.

### 3.2 Hemodynamic evaluation

#### 3.2.1 Hemodynamic evaluation of MVr

**Figure 2:**
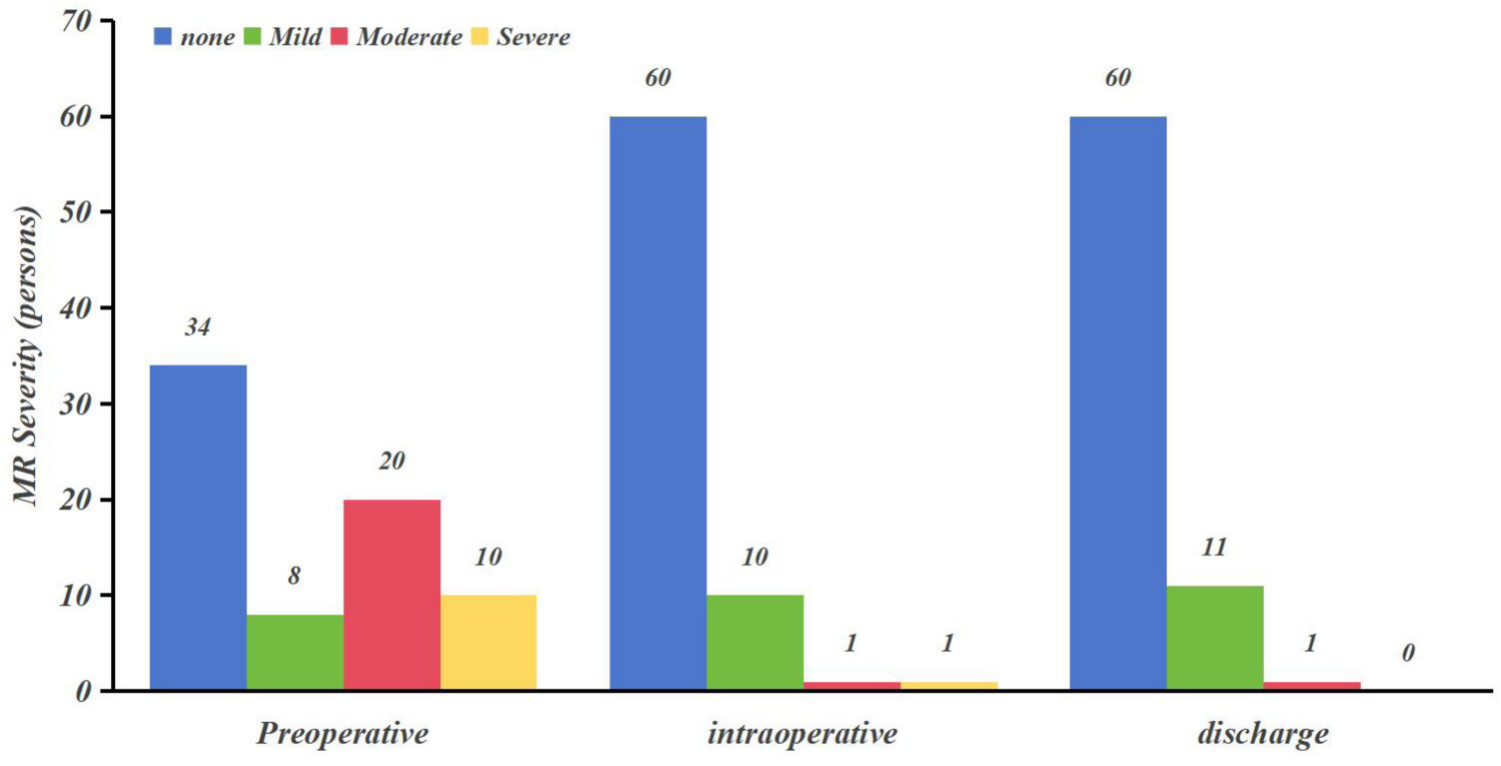
MR severity following MVr (preoperative vs. postoperative comparisons).

(1) Extent of mitral regurgitation (Table 2; Figure 2): There was a significant improvement in the level of mitral regurgitation in MVr patients intraoperatively and at the time of discharge compared with preoperatively (*P* < 0.05).
(2) Repeated-measures *ANOVA* demonstrated significant improvements in echocardiographic indices at all postoperative timepoints compared with baseline (Table 3): left ventricular end-diastolic diameters (LVEDD), left atrial end-systolic diameters (LAESD), mitral e-wave velocity, left ventricular ejection fraction (LVEF), mitral valve orifice area (MVOA), mitral pressure halving time (PHT), and mean pressure gradient (MPG) at each time point after MVr were improved compared with preoperative values (*P* < 0.05).

**Table 2:** MR severity following MVr (preoperative vs. postoperative comparisons) $\left[\bar{n}(\text{\%})\right]$.

MR severity	Preoperative	Intraoperative	Discharge
None	34 (47.20)	60 (83.30)	60 (83.30)
Mild	8 (11.10)	10 (13.90)	11 (15.30)
Moderate	20 (27.80)	1 (1.40)	1 (1.40)
Severe	10 (13.90)	1 (1.40)	0 (0)
*χ*2 *value*	–	31.968	34.856
*P-value*		<0.05^a^	<0.05^a^

^a^ *χ*^2^ test.

MR, mitral regurgitation.

**Table 3:** Echocardiographic outcomes following MVr (preoperative vs. postoperative comparisons) $\left[\bar{x}\pm s\right]$.

Variable	Preoperative	Time of discharge	3 months postoperatively	6 months postoperatively	*F*-value	*P-*value
LVEDD (mm)	45.81 ± 4.17	47.67 ± 5.27^a^^,^^c^^,^^d^	44.21 ± 2.39^a^^,^^b^^,^^d^	43.26 ± 1.60^a^^,^^b^^,^^c^	29.44	<0.05
LAESD (mm)	49.42 ± 5.43	43.07 ± 4.22^a^	42.47 ± 3.80^a^	41.75 ± 4.56^a^	93.37	<0.05
E-wave velocity (m/s)	2.03 ± 0.56	1.43 ± 0.37^a^	1.44 ± 0.27^a^	1.44 ± 0.23^a^	51.53	<0.05
LVEF (%)	53.89 ± 6.70	59.47 ± 5.25^a^^,^^c^	61.49 ± 4.09^a^^,^^b^^,^^d^	58.94 ± 4.68^a^^,^^c^	27.97	<0.05
LVEDV (ml)	113.6 ± 23.18	107.87 ± 26.67	108.17 ± 19.70	127.86 ± 159.90	0.74	<0.05
MVOA (cm^2^)	1.18 ± 0.43	2.41 ± 0.41^a^^,^^c^^,^^d^	2.33 ± 0.40^a^^,^^b^^,^^d^	2.23 ± 0.40^a^^,^^b^^,^^c^	309.38	<0.05
PHT (ms)	212.86 ± 88.33	95.46 ± 16.87^a^^,^^c^^,^^d^	99.56 ± 16.78^a^^,^^b^^,^^d^	104.42 ± 16.13^a^^,^^b^^,^^c^	123.59	<0.05
MPG (mmHg)	11.76 ± 6.42	3.21 ± 1.39^a^^,^^d^	3.35 ± 1.18^a^^,^^d^	3.83 ± 1.10^a^^,^^b^^,^^c^	118.5	<0.05

^a^ Comparison within group: compared with preoperative time point (*P* < 0.05).

^b^ Comparison within group: compared with discharge (*P* < 0.05).

^c^ Comparison within group: compared with 3-month postoperative time point (*P* < 0.05).

^d^ Comparison within group: compared with 6-month postoperative time point (*P* < 0.05).

LVEDD, left ventricular end-diastolic diameter; LAESD, end-systolic diameter of left atrium; LVEF, left ventricular ejection fraction; LVEDV, left ventricular end-diastolic volume; MVOA, mitral valve orifice area; PHT, pressure half-time; MPG, mean pressure gradient.

#### 3.2.2 Hemodynamic evaluation of MVr and MVR

(1) The results showed (see Table 4): At each postoperative time point (discharge, 3 months, and 6 months), the observation group demonstrated significantly lower LVEDD, LAESD, and mitral E-wave velocity than those in the control group (all *P* < 0.05).
(2) The observation group showed significantly lower LVPWd than that in the control group at both 3-month and 6-month postoperative follow-ups (*P* < 0.05).
(3) The observation group demonstrated significantly lower LVEF than that in the control group at 6-month postoperative follow-up (*P* < 0.05).
(4) The observation group had significantly lower LVEDV than that in the control group at 3-month postoperative follow-up (*P* < 0.05).

**Table 4:** Comparison of cardiac ultrasound indices between the two groups [$\bar{x}\pm s$, *n* (%), *M* (*P*_25_, *P*_75_)].

Variable	Groups	Preoperative	Time of discharge	3 months postoperatively	6 months postoperatively
LAESD (mm)	Control group	52.36 ± 6.25	46.53 ± 5.65	46.03 ± 4.51	44.67 ± 4.83
	Observation group	49.42 ± 5.43	43.07 ± 4.22	42.47 ± 3.80	41.75 ± 4.56
	*t*/*Z* value	3.182	5.865	5.386	2.233
	*P*-value	<0.05^a^	<0.05^a^	<0.05^a^	<0.05^a^
LVEF (%)	Control group	59.88 ± 7.20	60.43 ± 5.75	60.66 ± 5.54	60.76 ± 6.28
	Observation group	57.89 ± 6.70	59.47 ± 5.25	61.49 ± 4.09	58.94 ± 4.68
	*t*/*Z* value	5.384	1.098	−1.063	2.049
	*P*-value	>0.05^a^	>0.05^a^	>0.05^a^	<0.05^a^
LVEDV (ml)	Control group	115.96 ± 31.01	131 (113.25, 143.25)	115.67 ± 18.40	100.00 (88, 113)
	Observation group	107.87 ± 26.67	107.87 ± 26.67	108.17 ± 19.70	127.86 ± 159.90
	*t*/*Z* value	−1.803	−4.174	2.525	−4.6
	*P*-value	>0.05^a^	<0.05^b^	<0.05^a^	>0.05^b^
LVEDD (mm)	Control group	46.10 ± 5.17	49.97 ± 4.20	46.93 ± 3.56	44.66 ± 3.05
	Observation group	45.81 ± 4.17	47.67 ± 5.27	44.21 ± 2.39	43.26 ± 1.60
	*t*/*Z* value	−1.926	6.353	5.586	3.524
	*P*-value	>0.05^a^	<0.05^a^	<0.05^a^	<0.05^a^
LVPWd (mm)	Control group	9 (8,10)	10 (9,10)	10 (9,10)	10 (9,10)
	Observation group	10 (9,10)	9 (9,10)	9 (8,10)	9 (8,10)
	*t*/*Z* value	−1.03	−1.492	−4.529	−3.208
	*P*-value	>0.05^b^	>0.05^b^	<0.05^b^	<0.05^b^
E-wave velocity (m/s)	Control group	2.13 ± 0.49	1.8(1.50,2)	1.74 ± 0.42	1.67(1.4,1.99)
	Observation group	2.03 ± 0.56	1.43 ± 0.37	1.44 ± 0.27	1.44 ± 0.23
	*t*/*Z* value	1.269	−5.143	5.181	−4.482
	*P*-value	>0.05^a^	<0.05^b^	<0.05^a^	<0.05^b^

^a^ *t-*test.

^b^ Wilcoxon test.

LVEDD, left ventricular end-diastolic diameter; LAESD, end-systolic diameter of left atrium; LVEF, left ventricular ejection fraction; LVEDV, left ventricular end-diastolic volume; LVEDD, left ventricular end-diastolic diameters; LVPWd, left ventricular posterior wall thickness at end-diastole.

## 4 Discussion

RHD is one of the most common valvular diseases in cardiac surgery, characterized by leaflet thickening, diffuse fibrosis of the valve leaflets, and a “fish mouth”-shaped mitral orifice (12, 13). RHD predominantly affects the mitral valve, where persistent inflammatory reactions lead to valve thickening and commissural fusion, resulting in restricted leaflet motion and valvular stenosis. Additionally, it often involves the subvalvular apparatus, causing diffuse shortening and fusion of chordae tendineae. Clinically, this manifests as mitral stenosis (MS), mitral regurgitation (MR), or a combination of both (9). Due to geographical and economic constraints, rheumatic fever is relatively rare in developed countries, where mitral valve disease is primarily caused by degenerative changes leading to valve stenosis accompanied by leaflet thickening and calcification. In contrast, rheumatic heart disease resulting from rheumatic fever remains prevalent in China. The persistent inflammatory response leads to commissural fusion of the mitral valve leaflets, as well as shortening and adhesion of the subvalvular chordae tendineae (14, 15). Surgical intervention remains the primary treatment for organic mitral valve disease, primarily including PMBV, MVR, and MVr (16, 17). PMBV achieves commissural splitting and mitral valve dilation, resulting in low perioperative mortality, high success rates, and sustained patient satisfaction (18–20). However, the balloon catheter system may lead to severe complications such as cardiac tamponade, significant mitral regurgitation, and iatrogenic atrial septal defects (21). Usta et al. (22) conducted a 5-year follow-up of 276 rheumatic mitral stenosis patients who underwent PMBV and found reoperation rates of 5% and severe mitral regurgitation incidence of 15%. For patients with concomitant left atrial thrombus, severe mitral leaflet calcification, or rigidity, surgical intervention (MVr or MVR) is often required. Many rheumatic heart disease patients are young, and mechanical prostheses are often preferred for valve replacement. However, lifelong warfarin anticoagulation is mandatory after mechanical valve implantation, increasing the risks of thromboembolism and bleeding. These risks are significantly elevated in specific populations, such as pregnant women, and can substantially impact quality of life. Moreover, as the disease progresses, patients with prior MVR face an increased likelihood of requiring aortic valve surgery (18, 22). For those with contraindications to warfarin, bioprosthetic valve replacement is an alternative. However, this may necessitate reoperation due to structural valve deterioration, which is associated with higher mortality rates (23, 24). In contrast, MVr significantly improves left ventricular function by restoring the normal geometry, hemodynamics, leaflet mobility, and flexibility of the mitral valve. This approach reduces the incidence of infective endocarditis and thromboembolic events, lowers mortality rates, and enhances patients’ quality of life (25, 26).

### 4.1 Characteristics of rheumatic mitral valve disease

In this study, the proportions of female patients were 64.9% in the control group and 69.4% in the observation group, demonstrating a gender distribution pattern consistent with the well-established epidemiological characteristic of rheumatic mitral valve disease predominance in females. Rheumatic mitral valve disease in China often manifests as MS and/or MR, and in this study, there were 49 (52.13%) MS and 45 (47.87%) MS + MR in the control group, whereas there were 39 (54.17%) MS and 33 (45.83%) MS + MR patients in the observation group, which is consistent with the characteristics of rheumatic mitral valve lesions in China (27). The possible reasons analyzed are as follows: (1) rheumatic heart disease is an autoimmune disease, which is mainly associated with high levels of progesterone, estrogen, and corticosteroid hormones; (2) affected by rheumatoid inflammation, the valves become progressively calcified and fibrotic, with thickening of the valve leaflets, juxtaposition of adhesions, and contracture of the subvalvular structures; (3) the incidence rate is driven by resource scarcity, lack of antibiotics, and limited access to health care, but the incidence rate has been increasing with the continuous development of the economy, and the incidence rate tends to decrease.

### 4.2 Timing and strategies for MVr

The choice between rheumatic mitral valve repair and mitral valve replacement remains controversial despite continued scientific, technological, and surgical advances and improvements. With youth, the desire to become pregnant and poor compliance with mechanical valve anticoagulation therapy are important reasons for choosing rheumatic mitral valve repair. Patients with rheumatic mitral valve disease must be thoroughly evaluated for the extent and degree of mitral valve disease by cardiac ultrasound before deciding on the best treatment option. MVr is not appropriate from a durability standpoint if the patient has undergone previous mitral valve surgery or if the valve already has severe pathologic structural changes: severe calcification, fibrosis, and junctional fusion of the mitral leaflets, shortening of the tendons of the subvalvular structures, adhesions, and fibrosis of the papillary muscle cusps. If the mitral leaflets are still moderately mobile, mildly to moderately calcified, and have a sufficiently long tendon cord and poor compliance with anticoagulation therapy, then MVr may be considered. In this study, all patients in the observation group underwent comprehensive rheumatic mitral valve repair, including leaflet fibrous plaque decortication, commissurotomy, papillary muscle splitting, and annuloplasty. During fibrous plaque decortication, blunt dissection was initiated from the annular margin of the leaflet and progressively extended toward the free edge. This technique effectively restored leaflet pliability and native geometry while facilitating proper valve opening, thereby relieving leaflet restriction and achieving adequate orifice area. For mitral commissurotomy when subvalvular structures are poorly visualized, our protocol involves initial incision at the commissural area near the annular region and subsequent identification and meticulous management of the subvalvular apparatus. Moreover, shortened or elongated chordae tendineae may impair normal leaflet motion. Papillary muscle splitting serves as an effective approach to address subvalvular pathology, which establishes a competent valve orifice by optimizing chordal length and creating sufficient opening mobility. This comprehensive mitral valve repair technique demonstrates distinct differences from Professor Meng Xu's “Four-Step Approach.” It effectively eliminates calcific deposits from leaflets, relieves stenosis, restores native mobility and flexibility, and reconstructs physiological mitral geometry (28). Compared to valve replacement, these repair methods offer superior hemodynamic compatibility.

### 4.3 Hemodynamic evaluation of MVr

LVEDD, LAESD, mitral E-wave velocity, LVEF, MVOA, (PHT), and MPG at each time point after MVr were improved compared with preoperative values in this study. Rheumatic mitral stenosis reduces the effective area of the mitral valve orifice and decreases the positive blood flow from the left atrium to the left ventricle, leading to a decrease in left ventricular filling flow, which results in a decrease in the LVEDD and LVEDV compared with the normal state, and left ventricular equivalent to disuse atrophy. In addition, MS induces leaflet rigidity and restricted opening, resulting in persistently elevated left atrial-left ventricular pressure gradients that drive atrial dilatation and structural remodeling. After MVr, the patient's mitral stenosis is relieved, the leaflets regain their flexibility and geometry, the valve opens normally, the effective orifice area increases, and the left atrial flow to the left ventricle increases, resulting in left ventricular enlargement. At the same time, the left atrial blood flow resembles a “flooding” effect due to the left atrial–ventricular pressure gradient, resulting in a decrease in the LAESD at the end of systole (29). In addition, this study found that the degree of mitral regurgitation in MVr patients was significantly improved intraoperatively and at discharge compared with preoperatively (*P* < 0.05). The majority of patients who underwent MVr did not have significant postoperative MR (or only mild MR). Intraoperative TEE evaluation showed one case of moderate and one case of severe valvular regurgitation, which was intraoperatively repaired by our surgeon and discharged with only one case of moderate regurgitation. According to the 2020 ACC/AHA Guideline for the Management of Patients with Valvular Heart Disease (30), moderate or greater regurgitation of the valve after repair is considered poor or failed repair, and it is clearly stated that moderate or greater postoperative regurgitation is a Class IIa indication for reoperation (Level of Evidence B). The comprehensive mitral valve repair technique adopted in this study consists of four steps: debridement of calcified commissural plaques, peeling of fibrotic leaflet plaques, subcommissural incision and release, lysis of subvalvular papillary muscle adhesions, and implantation of an annuloplasty ring. The early success rate of this repair technique was 97.2%–98.6%, which was in line with the 2%–5% failure rate reported by Saurav et al. (31). However, the long-term failure rate of repair for rheumatic mitral valve disease remained high, with reoperation rates of up to 5%–10% within 5 years (32). The extent of MR was only retrospectively collected at two time points in this study, and further studies are needed to assess the results in the medium and even in the long term to follow up.

### 4.4 Hemodynamic evaluation of MVr and MVR

We compared and analyzed the hemodynamic parameters of the two groups at different time points and found the following significant results (*P* < 0.05). (1) Cardiac dimensional parameters: LVEDD, LAESD, and peak E velocity were significantly lower in the observation group than those in the control group at the time of discharge, 3 months after surgery, and 6 months after surgery. (2) Functional parameters: LVEF of the observation group was significantly lower than that in the control group at 6 months postoperatively, and LVPWd was significantly lower in the observation group at 3 and 6 months postoperatively. (3) Volumetric measurements: LVEDV was consistently lower in the observation group than that in the control group at the time of discharge and at 3 months postoperatively. These findings demonstrate superior hemodynamic improvements with MVr compared with MVR. The underlying mechanisms (33) may be explained as follows:

(1) Valve membrane preservation effect: MVr maintains the original valve device, restores the elasticity and mobility of the valve leaflets, and effectively relieves mitral stenosis.

(2) Hemodynamic changes:

(1) Stage 1 (early postoperative): The sudden release of the pressure gradient between the left atrium and the left ventricle produces a “floodgate” effect, resulting in a significant reduction of the left atrium, a moderate enlargement of the left ventricle.

(2) Phase II (3–6 month follow-up): The pressure gradient between the left atrium and left ventricle gradually returns to normal, and reverse cardiac remodeling begins to occur.

(3) Long-term benefits: restoration of near-physiologic cardiac function, maintenance of ventricular and annular continuity, and preservation of ventricular geometry.

### 4.5 Future outlook

Minimally invasive, rapid recovery, and excellent cosmetic results are the unremittingly pursued goals of future rheumatic mitral valve surgery. Yajima et al. (34) and other scholars used a robot to repair patients with rheumatic mitral valve lesions and found that patients recovered quickly after surgery and cardiac symptoms subsided. They also found that it was significantly better than conventional surgery in terms of fine manipulation, flexibility, and high-definition visualization and was effective in terms of cosmetic results and thoracic structural integrity. However, the robot lacked haptic feedback, and in the case of leaflet thickening and severe calcification, atrial rupture or coronary artery injury could occur. For individuals with healthy arteries and sufficient anterior mediastinal operating space, young people, or women with high aesthetic requirements, robotic mitral valve repair is feasible. Nowadays, the choice between rheumatic mitral valve repair and prosthetic valve replacement remains controversial. By comparing the hemodynamic changes of mitral valve repair and replacement, this study aims to evaluate the clinical value and application prospect of MVr and to provide more choices for the treatment of mitral valve lesions. However, there are limitations in this study: (1) The baseline data of the two groups of patients were incompletely collected, and there were some differences in the preoperative data, which could not achieve complete consistency in baseline like randomized controlled trials; (2) only the cardiac ultrasonography results of the patients were collected for 6 months after the operation, and the collection of evaluation indexes reflecting the efficacy of the operation was incomplete; (3) the number of included cases was small, and the follow-up period was short, and no statistics were kept on the perioperative complications and the quality of life in the postoperative period; (4) only a few postoperative time points of ultrasound results, which can approximate the short-term effect of surgery, and the medium- and long-term effects still need further study.

## 5 Conclusions

Mitral valve repair (MVr) and mitral valve replacement (MVR) are effective in the treatment of patients with rheumatic mitral valve disease, with MVr having superior short-term clinical outcomes than MVR.

## Data Availability

The datasets presented in this study can be found in online repositories. The names of the repository/repositories and accession number(s) can be found in the article/Supplementary Material.
